# Phototherapy for Cognitive Function in Patients With Dementia: A Systematic Review and Meta-Analysis

**DOI:** 10.3389/fnagi.2022.936489

**Published:** 2022-06-30

**Authors:** Genying Zhu, Qifeng Tong, Xiangming Ye, Juebao Li, Liang Zhou, Peng Sun, Feng Liang, Shuchang Zhong, Ruidong Cheng, Jie Zhang

**Affiliations:** ^1^Center for Rehabilitation Medicine, Rehabilitation & Sports Medicine Research Institute of Zhejiang Province, Department of Rehabilitation Medicine, Zhejiang Provincial People’s Hospital (Affiliated People’s Hospital, Hangzhou Medical College), Hangzhou, China; ^2^Department of Neurology & Brain Medical Centre, The First Affiliated Hospital, Zhejiang University School of Medicine, Hangzhou, China; ^3^College of Rehabilitation, Zhejiang Chinese Medical University, Hangzhou, China

**Keywords:** dementia, phototherapy, cognition, older people, light, meta-analysis

## Abstract

**Background:**

Dementia is a major health burden worldwide. As numerous pharmacological trials for dementia have failed, emerging phototherapy studies have evaluated the efficacy of alternative therapies for cognition.

**Objective:**

The objective of this study was to evaluate the association between phototherapy and changes in cognitive deficits in patients with dementia.

**Methods:**

PubMed, Embase, Web of Science, PsycINFO, CINAHL, and Cochrane Central Register of Controlled Trials were searched from inception to 27 March 2022. Inclusion criteria were controlled clinical trials of phototherapy interventions reporting pre-post changes in global cognitive function and subdomains in patients with dementia. Data were extracted by two independent reviewers and pooled in random-effects models. Subgroup and meta-regression analyses were conducted to investigate the sources of heterogeneity.

**Results:**

Our analyses included 13 studies enrolling a total of 608 participants. Phototherapy showed significant associations with improvements of global cognitive function (standardized mean difference [SMD], 0.63; 95% confidence interval [CI], 0.33–0.94; *P* < 0.001) and subdomains, especially with respect to attention, executive function, and working memory. Near-infrared (NIR) light-emitting diodes (LEDs) photobiomodulation (SMD, 0.91; 95% CI, 0.46–1.36; *P* < 0.001) and lasers (SMD, 0.99; 95% CI, 0.56–1.43; *P* < 0.001) showed more significant associations with improved cognitive functions when compared with normal visible light. In addition, the effect sizes of short-term effects (SMD, 0.63; 95% CI, 0.33–0.94; *P* < 0.001) were larger than effects assessed in long-term follow-up (SMD, 0.49; 95% CI, -0.24–1.21; *P* = 0.189).

**Conclusion:**

In this meta-analysis, phototherapy interventions were associated with cognitive improvement in patients with dementia. NIR LEDs and lasers had advantages over normal visible light. Domain-specific effects were indicated for attention, executive function, and working memory. Short-term improvement after phototherapy was supported, while evidence for long-term benefits was lacking. Stronger evidence for individualized parameters, such as optimal dosing, is needed in the future.

**Systematic Review Registration:**

[https://www.crd.york.ac.uk/prospero/display_record.php?RecordID=267596], identifier [CRD42021267596].

## Introduction

With a growing aging population, dementia has become a major global health burden. Over 55 million patients worldwide are diagnosed with dementia (Gauthier et al., 2021), and this number is estimated to triple in 30 years (Alzheimer’s Disease International, 2019). Given that breakthrough treatments and preventive interventions are lacking, the effective management of dementia is highly challenging. Moreover, an increased risk of inappropriate prescribing may lead to higher rates of adverse health outcomes (Delgado et al., 2021).

Dementia is an umbrella term covering heterogeneous causes of neurocognitive disease. Alzheimer’s disease (AD) is the most prevalent type of dementia, accounting for 60–70% of cases, followed by vascular dementia (VD) and Lewy body dementia (Burns and Iliffe, 2009). Regardless of the dementia subtype, the quality of life of patients and the sociopsychological conditions of their families are adversely affected. Cognitive decline is the core symptom of dementia and can undermine independent activities of daily living (Muir et al., 2012). For instance, loss of short-term memory occurs in the early stages of AD; in later stages, executive, visuospatial, and language abilities are frequently impaired (Atri, 2019). VD presents predominantly with symptoms in the subdomains of executive function and processing speed (Iadecola et al., 2019).

As numerous pharmacological trials in dementia have failed, a rising number of researchers have turned to investigate the potential benefits of non-pharmacological interventions (Liu et al., 2020; Wang et al., 2020; Sikkes et al., 2021). Phototherapy may be a novel non-pharmacological intervention for improving the cognitive symptoms of dementia. The light types of phototherapy can be broadly categorized into bright and near-infrared (NIR) light and can be more finely divided into light-emitting diodes (LEDs) and lasers (Enengl et al., 2020). Photobiomodulation (PBM), an emerging technology, refers specifically to neuromodulation via transcranial phototherapy, which is different from the conventional light therapy mediated by environmental changes (Hamblin, 2019). However, there is a lack of solid evidence from highly powered investigations and pooled results of rigorously designed clinical studies (Salehpour et al., 2021).

A previous meta-analysis regarding phototherapy for dementia merely pooled post-therapy cognitive scores from three studies (Forbes et al., 2014). Detailed cognitive subdomains and influences of moderators were not investigated in this previous review. Moreover, it was published 8 years before and thus did not include a growing body of recent literature. Therefore, conducting an updated meta-analysis is well advised to examine the associations between phototherapy and cognitive improvement in dementia and also ascertain potential influencing and confounding factors.

## Methods

The protocol for this systematic review and meta-analysis was registered on PROSPERO (CRD42021267596). This study was reported according to the Preferred Reporting Items for Systematic Reviews and Meta-Analyses (PRISMA) guidelines (Page et al., 2021).

### Search Strategy

A systematic literature search was performed using electronic databases (PubMed, Embase, Web of Science, CINAHL, PsycINFO, and Cochrane Central Register of Controlled Trials [CENTRAL]) from inception to 27 March 2022. The search strategy included synonyms of major keywords and medical subject headings. The AND operator was used to combine the search results with regard to the target patients (dementia), intervention (phototherapy), and study design (controlled trial) (Supplementary Table 1). The language was not restricted, and the reference lists of all relevant studies in previous reviews were checked to avoid potential omissions.

### Selection Criteria

Two reviewers (G.Z. and J.Z.) independently screened the titles and selected relevant studies according to the abstracts. The eligibility of the screened articles was checked by a full-text review. The selection criteria were as follows: (1) participants: dementia patients (with an average age of > 60 years) were included in this study (the etiology underlying dementia was not specified, including AD, VD, and mixed dementia); (2) interventions: phototherapy, also known as light therapy, utilized normal visible light (traditional bright light and blue-enriched light), NIR LED, or lasers; (3) comparators: the interventions administered to the control groups could be placebos, sham stimulation, or conventional care; (4) outcomes: global cognitive function and subdomains were measured by cognitive scales including the Mini-Mental State Examination and the Montreal Cognitive Assessment; and (5) studies: we limited the eligible study type to controlled trials for reliable results.

### Data Extraction

Two reviewers (Q.T. and J.Z.) independently extracted the following demographic and social information from the eligible publications. Study characteristics and intervention-related features were collected. Outcome data, including scale scores for each intervention group, were extracted from a pre-designed electronic sheet. The Engauge Digitizer software (version 12.1) was used to extract data from studies that only rendered figures instead of directly accessible values (Mitchell et al., 2022). The number of reasons for dropouts as well as adverse events was recorded.

### Risk of Bias

The risk of bias for all the included studies was assessed by two independent reviewers (G.Z. and Q.T.) using the Cochrane Collaboration’s tool (Risk of Bias version 2 [RoB2], London, United Kingdom) (Sterne et al., 2019). Discrepancies between the two reviewers were mediated by a third reviewer (J.Z.). Judgments for the overall risk of bias according to the algorithms provided by the RoB2 tool were calculated automatically based on five bias domains.

### Statistical Analyses

Meta-analyses were conducted using Stata MP version 17.0 (StataCorp, College Station, TX, United States). All data evaluated for quantitative merging were continuous outcomes in the form of mean change values and their standard deviations. Standard deviation values were estimated for trials that reported pre- and post-therapy scores without change values, as instructed by the Cochrane Handbook for Systematic Reviews of Interventions (Higgins et al., 2019). Considering various cognitive function scales, we chose standardized mean differences (SMDs) and 95% confidence intervals (CIs) to evaluate effect sizes. Estimated SMDs were then pooled together using a random-effects model via the inverse variance method. The level of statistical heterogeneity was measured using the *I*^2^ statistic, and substantial heterogeneity was defined as values of > 50%. A sensitivity analysis was performed excluding studies with a high risk of bias to check their potential contribution to heterogeneity. The threshold for a statistically significant effect size was set at *P* < 0.05.

Subgroup analyses were conducted to identify the sources of heterogeneity and inter-subgroup differences in effect sizes. Multiple subgroup categories were pre-specified as follows: (1) time of assessment; (2) cognitive subdomains; (3) phototherapy subtypes comprising normal visible light (traditional bright light and blue-enriched light), NIR LED, and laser; and (4) types of dementia. Meta-regression analyses were performed to determine the influence of moderators including mean age, female sex, education, area, etiology, and intervention characteristics. Univariate meta-regression models for the above moderators were analyzed using Stata MP.

Funnel plots and Egger’s tests were used to assess the potential publication bias for outcomes evaluated in more than 10 studies. Publication bias was considered significant if the funnel plot was asymmetric or if the *P-*value of Egger’s test was < 0.1.

## Results

**FIGURE 1:**
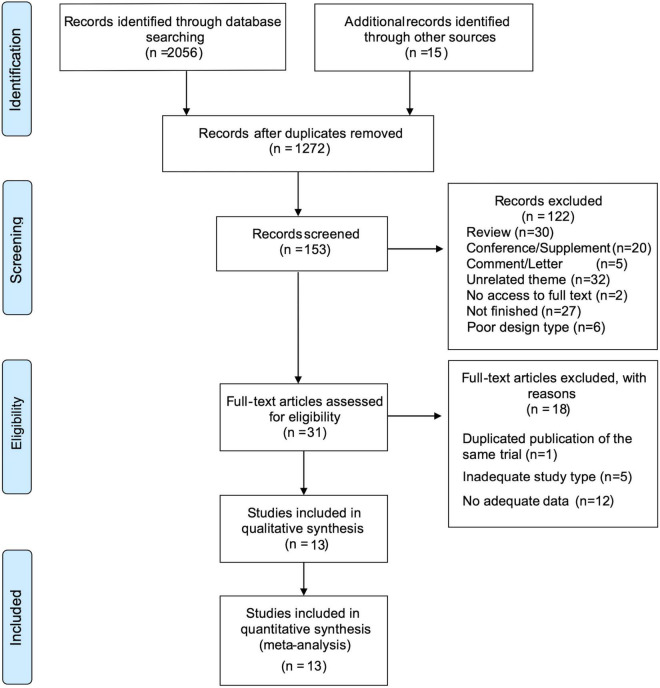
PRISMA flow diagram depicting the study process. n, the number of records; PRISMA, Preferred Reporting Items for Systematic Reviews and Meta-Analyses.

We collected 2,056 records from six databases and identified 15 records from other sources, including reference lists from previous relevant reviews. After removing duplicates, 1,272 items remained. We identified 153 records via title screening, and the abstracts of these records were screened more comprehensively. A total of 31 articles were selected for a full-text review, and 13 articles were considered eligible for quantitative meta-analysis. The details of excluded trials are shown in Figure 1.

### Study Characteristics

The characteristics of the included studies are summarized in Supplementary Table 2. All 13 trials included in this meta-analysis were controlled studies, and 10 were randomized controlled trials. Among these, three trials were performed in the United States (Berman Marvin et al., 2017; Chao, 2019; Nizamutdinov et al., 2021), five were performed in Asia (Guo et al., 1998; Huang et al., 2015; Chan et al., 2021; Kim et al., 2021; Liu et al., 2021), four were performed in Europe (Graf et al., 2001; Riemersma-van der Lek et al., 2008; Burns et al., 2009; Cremascoli et al., 2022), and one was performed in Egypt (Nagy et al., 2021).

A total of 608 participants were enrolled; the number of participants in each individual study varied from *n* = 8 to *n* = 143. The mean age of participants in each group ranged between 63.9 and 85.8 years, and the proportion of women varied from 30.8 to 92.3%. The mean education level of the elderly participants ranged from 3.8 to 18.3 years. Regarding the etiology of dementia, seven studies only included patients with AD (Graf et al., 2001; Huang et al., 2015; Berman Marvin et al., 2017; Chao, 2019; Kim et al., 2021; Nagy et al., 2021; Cremascoli et al., 2022), one study included only patients with VD (Guo et al., 1998), and the remaining five studies included patients with mixed types of dementia (Riemersma-van der Lek et al., 2008; Burns et al., 2009; Chan et al., 2021; Liu et al., 2021; Nizamutdinov et al., 2021). Regarding dementia severity, most studies reported mild to moderate dementia.

The evaluated phototherapy interventions could be categorized into three subtypes, namely, normal visible light (Graf et al., 2001; Riemersma-van der Lek et al., 2008; Burns et al., 2009; Huang et al., 2015; Kim et al., 2021; Liu et al., 2021; Cremascoli et al., 2022), NIR LED PBM (Berman Marvin et al., 2017; Chao, 2019; Chan et al., 2021; Nizamutdinov et al., 2021), and laser (Guo et al., 1998; Nagy et al., 2021). Normal visible light could be further categorized into traditional bright light and blue-enriched light. The phototherapy duration of each session varied from 350 s to 9 h, while the frequency ranged from 3 to 14 sessions per week. The activities during a session of phototherapy included sitting still, talking, reading, listening to music, or a daily group session, and most studies did not provide details about any specific activity. Other intervention- and measurement-associated features are listed in Supplementary Tables 3, 4.

### Risk of Bias

The risk of bias for individual studies and the percentage graph depicting the risk of bias summary are shown in Supplementary Figure 1. Among the 13 included studies, a high risk of overall bias was detected in three investigations, and two studies were rated as low risk. The remaining eight studies (61.5%) exhibited some concerns regarding overall bias. With respect to risk of bias domains, three studies reported a high risk of bias in the randomization process (Guo et al., 1998; Kim et al., 2021; Liu et al., 2021). The majority of the studies exhibited a low risk of bias in the three evaluated domains, namely, missing outcome data, outcome measurement, and selective reporting.

### Effects of Interventions on Cognition

**FIGURE 2:**
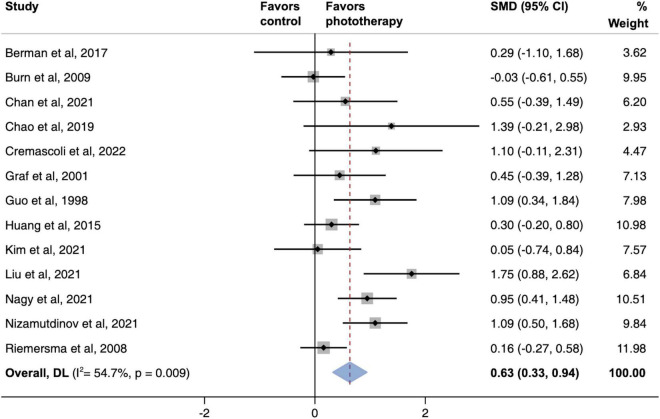
Pooled effects of global cognitive function changes after phototherapy as compared to control interventions. SMD, standardized mean difference. Weights are from random-effects model.

The global severity of cognitive defects was assessed using the Mini-Mental State Examination in most studies. The meta-analysis of this primary outcome showed that phototherapy was associated with significantly improved global cognitive function (SMD, 0.63; 95% CI, 0.33–0.94; *P* < 0.001) as compared with the control group (Figure 2). The pooled results exhibited substantial heterogeneity (*I*^2^ = 54.7%, *P* = 0.009). A sensitivity analysis excluding studies with a high risk of bias demonstrated a similar result (SMD, 0.58; 95% CI, 0.29–0.87; *P* = 0.001) that reduced the degree of heterogeneity (*I*^2^ = 42.9%; *P* = 0.063) (Supplementary Figure 2).

**FIGURE 3:**
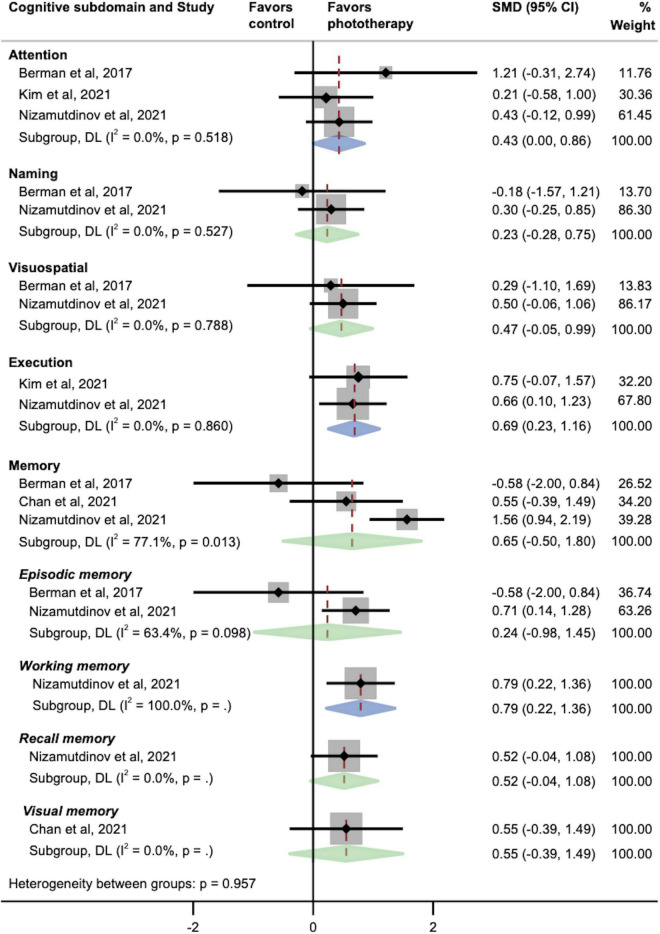
Pooled effects of different cognitive subdomains after phototherapy as compared with control interventions. SMD, standardized mean difference. Weights and between-subgroup heterogeneity test are from random-effects model.

The pooled effects of multiple cognitive subdomains were investigated as well (Figure 3). We found that phototherapy was associated with significant increases in the subdomains of attention (SMD, 0.43; 95% CI, 0.00–0.86; *P* = 0.05), executive function (SMD, 0.69; 95% CI, 0.23–1.16; *P* = 0.004), and working memory (SMD, 0.79; 95% CI, 0.22–1.36; *P* = 0.006). In contrast, there were no statistically significant improvements in the overall memory domain and its subcomponents except working memory, including episodic memory, recall memory, and visual memory. Similarly, the pooled results of naming and visuospatial abilities did not exhibit larger effect sizes in the phototherapy group as compared with the placebo group.

### Subgroup Analyses

**FIGURE 4:**
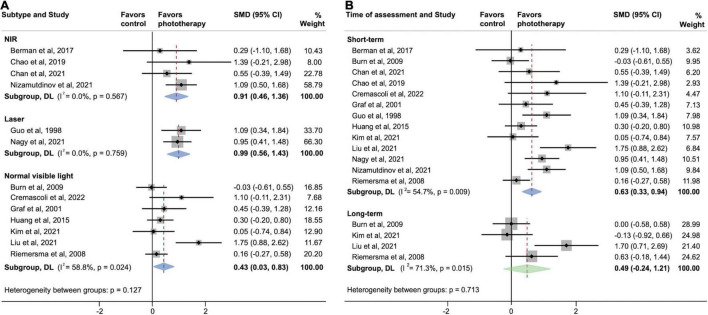
Subgroup analyses evaluating cognitive improvement, stratified by **(A)** phototherapy subtypes and **(B)** time of assessment. NIR, near-infrared; SMD, standardized mean difference. Weights and between-subgroup heterogeneity test are from random-effects model.

**FIGURE 5:**
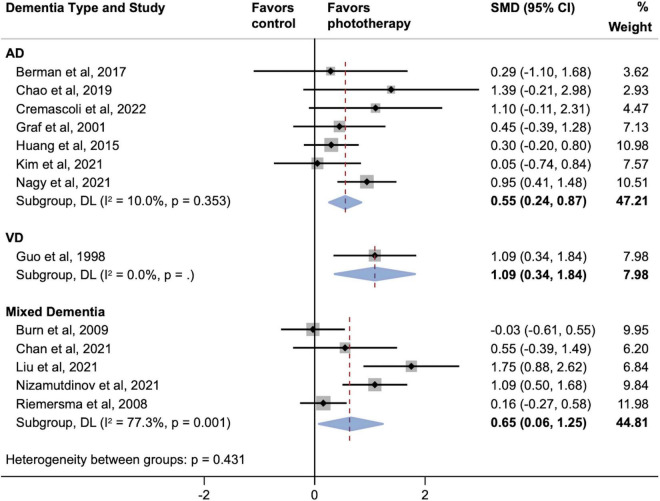
Subgroup analyses evaluating cognitive improvement, stratified by types of dementia. AD, Alzheimer’s disease; SMD, standardized mean difference; VD, vascular dementia. Weights and between-subgroup heterogeneity test are from random-effects model.

Figure 4 shows two parallel subgroup analyses investigating the source of heterogeneity in the primary outcome. As shown in Figure 4A, subgroups defined according to phototherapy subtypes dramatically reduced heterogeneity in subgroups of NIR LED PBM and laser (both *I*^2^ = 0.0%; *P* > 0.05). Both NIR LED PBM (SMD, 0.91; 95% CI, 0.46–1.36) and laser (SMD, 0.99; 95% CI, 0.56–1.43) interventions yielded statistically significant effects on cognitive improvement (both *P* < 0.001), while the pooled effect size of normal visible light interventions was smaller (SMD, 0.43; 95% CI, 0.03–0.83; *P* = 0.034). Further analyses on subtypes of normal visible light interventions did not detect inter-subgroup heterogeneity (*P* = 0.953). A subgroup analysis according to time of assessment showed that short-term effects post-phototherapy (SMD, 0.63; 95% CI, 0.33–0.94; *P* < 0.001) was stronger than effects assessed during long-term follow-up (SMD, 0.49; 95% CI, -0.24–1.21; *P* = 0.189) (Figure 4B). In addition, subgroup analysis by types of dementia showed significant associations between phototherapy interventions and improved cognitive functions in AD (SMD, 0.55; 95% CI, 0.24–0.87; *P* = 0.001), VD (SMD, 1.09; 95% CI, 0.34–1.84; *P* = 0.004), and mixed dementia (SMD, 0.65; 95% CI, 0.06–1.25; *P* = 0.032) after phototherapy (Figure 5). The sensitivity analyses for the subgroup analyses are shown in Supplementary Figures 3–5. By excluding studies with a high risk of bias, only the pooled effect sizes of normal visible light interventions (SMD, 0.23; 95% CI, -0.03 to 0.49; *P* = 0.083) and mixed dementia (SMD, 0.42; 95% CI, −0.10 to 0.93; *P* = 0.111) were decreased and showed trend-level efficacy.

### Moderator Analysis

The results of univariate meta-regression analyses for multiple moderators in all the included 13 studies are summarized in Table 1. All of the moderators, including mean age, sex, education, area, etiology, and intervention characteristics, did not have any statistically significant or trend-level associations with global cognitive improvement (*P* > 0.05). As shown in Table 2, further meta-regression analyses in the studies using normal visible light also detected no significant associations between cognitive improvement and light features, including subtypes (traditional bright light vs. blue-enriched light), intensity of illumination, and circadian rhythm.

**TABLE 1:** Summary of univariate meta-regression analyses for the effects on global cognitive function.

Moderators	Coefficient (95% CI)	*P-*values
*Demographics*		
Mean age (years)	−0.031 (−0.087, 0.026)	0.259
Sex (female%)	−1.386 (−3.540, 0.768)	0.184
Education level (years)	0.027 (−0.073, 0.127)	0.561
Area		
-American	0.030 (−1.391, 1.452)	0.963
-Europe	−0.200 (−1.160, 0.760)	0.648
-Asia	−0.176 (−1.116, 0.764)	0.682
*Etiology of dementia*		
-AD	−0.309 (−1.304, 0.685)	0.504
-Mixed	−0.333 (−1.411, 0.745)	0.507
*Intervention characteristics*		
Duration of each session (min)	−0.003 (−0.008, 0.002)	0.217
Frequency of sessions (day/week)	−0.010 (−0.125, 0.105)	0.852
Length of therapy period (day)	−0.008 (−0.004, 0.021)	0.174
Type of photobiomodulation		
-Normal visible light	−0.122 (−0.872, 0.627)	0.724
-Laser	0.211 (−0.715, 1.137)	0.623

*AD, Alzheimer’s disease; CI, confidence interval.*

**TABLE 2:** Summary of univariate meta-regression analyses for global cognitive function in relationship to normal visible light.

Moderators	Coefficient (95% CI)	*P-*values
*Features of light*		
Subtypes of normal visible light -Traditional bright light vs. blue-enriched light	0.484 (−0.590, 1.558)	0.299
Intensity of illumination (lux)	−0.000 (−0.000, 0.000)	0.314
Circadian rhythm		
-Individualized by DLMO	0.629 (−3.145, 4.403)	0.633
-Morning	0.403 (−3.363, 4.168)	0.756
-Afternoon	0.291 (−3.988, 4.570)	0.842
*Demographics*		
Mean age (years)	−0.068 (−0.194, 0.059)	0.227
Sex (female%)	−1.387 (−4.067, 1.293)	0.241
Education level (years)	0.055 (−0.195, 0.306)	0.595
Area	0.055 (−1.345, 1.455)	0.924
*Etiology of dementia*	0.063 (−1.416, 1.542)	0.917
*Intervention characteristics*		
Duration of each session (min)	−0.002 (−0.007, 0.002)	0.253
Frequency of sessions (day/week)	−0.610 (−1.760, 0.542)	0.232
Length of therapy period (day)	0.030 (−0.030, 0.090)	0.253

*CI, confidence interval; DLMO, dim light melatonin onset.*

### Publication Bias

Neither evaluations of the symmetry of the funnel plot (Supplementary Figure 6) nor the results of Egger’s test (*P* = 0.199) detected any significant publication bias across the 13 included studies. Therefore, we concluded that the probability that the effect size of phototherapy interventions was influenced by publication bias was low.

## Discussion

To the best of our knowledge, this is the first meta-analysis to focus on cognitive changes after phototherapy in patients with dementia and to elaborate on domain-specific effects across global cognition, attention, executive function, memory, naming, and visuospatial abilities. This meta-analysis merged the results of 13 controlled trials (including 10 randomized controlled trials) investigating the associations between phototherapy interventions and cognitive improvement in patients with dementia. The pooled results from the 608 enrolled participants provided solid evidence for the benefits of phototherapy with respect to global cognitive function. Subdomain-specific variations of effect sizes were also detected. Moreover, this review provided a detailed summary of moderators, showing the influence of demographics, time of assessment, and intervention parameters on pooled therapeutic effects.

### Phototherapy Effects and Underlying Mechanisms

Considering that phototherapy comprises a generalized range of light-associated interventions, our study revealed varying effect sizes across different phototherapy subtypes. NIR LED PBM and laser interventions showed larger effect sizes in alleviating cognitive deficits as compared with normal visible light. The potential fundamental underlying neurobiological phototherapy modulations for these modalities are similar. They engage cell-based chromophores and trigger downstream molecules and biochemical pathways in the mitochondrial respiratory chain to exert a therapeutic effect (Karu, 2008; Hamblin, 2018; Wei et al., 2020). In addition, PBM can elevate perfusion levels by regulating regional brain blood flow (Salgado et al., 2015; Chao, 2019). Moreover, inhibition of amyloid development and amyloid beta (Aβ)-induced nerve cell apoptosis have been observed in animal and *in vitro* (non-human) experiments using PBM (De Taboada et al., 2011; Liang et al., 2012; Zhang et al., 2012). Given shared fundamental mechanisms, the varying effects of phototherapy may depend on the specific light parameters influencing the transcranial penetration ability of each modality. Compared with bright light, the wavelengths in the red and NIR regions that are used in transcranial PBM are more suitable for neuromodulation, with higher doses leading to deeper penetration depths and resulting in stronger neurobiological responses (Wang and Li, 2019). Compared with lasers, LEDs have equally effective performance at substantial levels of power density, with concomitant advantages in safety and cost (Heiskanen and Hamblin, 2018). Future trials should investigate the effects of phototherapy more comprehensively to improve the selection and implementation of light-associated parameters.

### Varying Effects Across Cognitive Subdomains

Apart from global cognitive function, our study supported domain-specific effects in terms of cognitive improvements in attention, executive function, and working memory, while no improvement was indicated in the other subdomains of memory, naming, and visuospatial abilities. Given the lack of effects on episodic memory combined with the time-dependent improvement, phototherapy tends to be a symptomatic rather than disease-modifying therapy, without producing an enduring change in the clinical progression and underlying mechanism of cell death (Cummings and Fox, 2017). Although the associations between phototherapy and cognition do not seem to cover all subdomains, it is inspiring to observe improvements in working memory and executive function that are pivotal to intellect and cognitive abilities (Nee et al., 2013).

The three domains associated with phototherapy exist in a complex relationship. A prevailing theory regards working memory as one of the three core executive functions determining the performance of complex executive tasks (Miyake et al., 2000; Diamond, 2013), while executive processes are critical components of working memory (Nee et al., 2013). Moreover, selective attention subserves working memory by selective information processing and storage (Awh and Jonides, 2001; Kiyonaga and Egner, 2013). In addition, they are among the most sensible cognitive domains for alertness and alerting effects. The alerting network, along with executive control and orienting, constitutes partially distinct networks supporting attention (Petersen and Posner, 2012). Therefore, the effects of phototherapy might be associated with changes in the alerting network, which supports achieving and keeping an alerting state (de Souza Almeida et al., 2021). Given that these three cognitive subdomains share common neuropsychological processes, it is plausible to observe simultaneous benefits due to these interventions after phototherapy. The underlying neural correlates of the phototherapy effects might be related to the functional regulations on the frontoparietal regions, which are exactly the critical areas for the domains of attention, executive function, and working memory (Head et al., 1999; Andres, 2003; Baldo and Dronkers, 2006; Scolari et al., 2015). Considering that dementia patients might have cognitive symptoms in varying subdomains, comprehensive neuropsychological investigations are needed to optimize individualized phototherapy.

### Time-Dependent Efficacy

Although the heterogeneity between short- and long-term assessments was insignificant in this study, the subgroup analysis by time of assessment supported the immediate effects of phototherapy on cognition. However, the lasting efficacy of this modality might be limited. A previous meta-analysis evaluating the efficacy of phototherapy for ameliorating dementia merely reported pooling cognition effects after 10–42 days of treatment (Forbes et al., 2014). Currently, there is a lack of sufficient evidence regarding time–response relationships based on animal studies, *in vitro* experiments, and clinical investigations.

### Influence of Moderators

Socioeconomic factors, including age, sex, and education, could be associated with cognitive reserve and functions, which may potentially influence the efficiency of interventions (Jack, et al., 2015; Russ, 2018; Zaninotto et al., 2018). However, our analysis suggested that none of the demographic and intervention-associated factors were potential moderators influencing the effects of phototherapy on cognitive improvement. Moreover, decreased amplitude and robustness of circadian activity rhythm along with delayed rhythm might be the underlying mechanisms of cognitive decline (Maiese, 2021) and might be associated with an increased risk of developing dementia and mild cognitive impairment (Tranah et al., 2011). Although the circadian rhythm has emerged as a critical factor for patients with dementia (Slats et al., 2013; Kumar et al., 2022), our meta-regression did not show preferred recommendations among morning, afternoon, or individualized rhythm. Levels of cognitive impairment have also been found to correlate with behavioral and psychological symptoms including apathy, aggressive behavior, and aberrant motor behavior (Lovheim et al., 2008), while reductions of abnormal behaviors in severe stages were reported by other researchers (Holtzer et al., 2003). In addition, the potential influence of different latitudes along with varying sun-light exposure on the effects of phototherapy remains unclear, due to less evidence from the current individual studies.

### Limitations

This study has several limitations. The number of phototherapy trials conducted in human dementia is small, and there are insufficient studies regarding phototherapy subtypes and subdomains of cognition. The overall effects and conclusions derived from subgroup analyses would be more statistically convincing with more phototherapy trials, and we strongly recommend that trials overcome this issue in the future. Second, the overall heterogeneity observed in this study was considerable, which may limit the interpretability of the conclusions; therefore, we have conducted detailed sensitivity analyses, subgroup analyses, and meta-regressions to determine the source of any heterogeneity. We also note that the variety of cognitive scales evaluated in this review might introduce measurement heterogeneity, although we calculated SMD values to reduce the potential influence of this concern. Although some of the included studies also assessed sleep and behavior disorders, their influence was not considered in the moderator analyses because of incomplete data and should be further analyzed in the future. In addition, the impact of dementia severity was not investigated because of insufficient relevant information available across the literature. Several phototherapy trials have reported mild to moderate levels of dementia severity, but the differences in therapeutic response within mild, moderate, and severe dementia remain uncertain.

## Conclusion

Phototherapy is a promising non-pharmacological option associated with cognitive improvement in patients with dementia. This meta-analysis suggests that NIR LED PBM and laser therapy may have advantages over normal visible light in terms of the associations with cognitive improvements in dementia. Domain-specific effects were statistically significant for attention, executive function, and working memory. The association between phototherapy and short-term cognitive improvement is supported, while the benefits in long-term follow-up seem to be limited. Optimal dosing and population-specific effects with regard to dementia severity and other modulating factors need to be clarified for informing the effective administration of precise individualized phototherapy in the future.

## Data Availability Statement

The original contributions presented in this study are included in the article/Supplementary Material, further inquiries can be directed to the corresponding author.

## Author Contributions

JL and XY were involved in the study concept and design. GZ and JZ contributed to literature searches, study selection, and data extraction. GZ, QT, and JZ assessed the quality of studies and responsible for drafting the manuscript. JL, RC, PS, FL, and SZ interpreted the data and revised the manuscript. RC, LZ, and SZ conducted the statistical analysis. XY, LZ, and JZ contributed to supervision. All authors reviewed and approved the final version of the manuscript.

## Conflict of Interest

The authors declare that the research was conducted in the absence of any commercial or financial relationships that could be construed as a potential conflict of interest.

## Publisher’s Note

All claims expressed in this article are solely those of the authors and do not necessarily represent those of their affiliated organizations, or those of the publisher, the editors and the reviewers. Any product that may be evaluated in this article, or claim that may be made by its manufacturer, is not guaranteed or endorsed by the publisher.
