# Supplementary material for: Phototherapy for Cognitive Function in Patients With Dementia: A Systematic Review and Meta-Analysis
Source: Front Aging Neurosci. 2022 Jun 30;14:936489. doi: 10.3389/fnagi.2022.936489 (PMC9284896; doi:10.3389/fnagi.2022.936489)
Supplement: Supplementary file 1 [file Data_Sheet_1.PDF]

Supplementary Material

1 Supplementary Tables

Supplementary Table 1. Literature search strategy in different electronic databases

Database	Time	Strategy	Number of Records
**Pubmed**	**2022.03.27**	#1 dementia[Title/Abstract] OR Alzheimer[Title/Abstract] OR Amnestic[Title/Abstract] OR Amnesia[Title/Abstract] OR "mild cognitive impairment"[Title/Abstract] OR "Dementia"[Mesh] OR "cognitive dysfunction"[Mesh]<br>#2 photobiomodulation[Title/Abstract] OR phototherapy[Title/Abstract] OR photoneuromodulation[Title/Abstract] OR photo-neuro-modulation[Title/Abstract] OR "light therapy"[Title/Abstract] OR "near infrared"[Title/Abstract] OR "near-infrared"[Title/Abstract] OR laser[Title/Abstract] OR "light emitting diode"[Title/Abstract]<br>#3 "randomized controlled trial"[Publication Type] OR "controlled clinical trial"[Publication Type] OR randomized[Title/Abstract] OR randomised[Title/Abstract] OR randomly[Title/Abstract] OR placebo[Title/Abstract] OR "double-blind"[Title/Abstract] or trial[Title/Abstract] OR groups[Title/Abstract] OR "controlled study"[Title/Abstract] OR RCT[Title/Abstract] OR "single-blind*"[Title/Abstract]<br>#4 #1 AND #2 AND #3	**270**

**Web of Science**	**2022.03.27**	#1 TS=dementia OR Alzheimer OR amnestic OR amnesia OR "mild cognitive impairment" OR "cognitive dysfunction"<br>#2 TS=photobiomodulation OR phototherapy OR photoneuromodulation OR photo-neuro-modulation OR "light therapy" OR "near infrared" OR "near-infrared" OR laser OR "light emitting diode"<br>#3 TS=randomised OR randomized OR randomly or placebo or "double-blind" or trial OR groups OR "controlled study" OR RCT OR "single-blind*"<br>#4 Refined By: Document Types: Review Articles OR Meeting OR Patent OR Abstract OR Editorial Materials<br>#5 #1 AND #2 AND #3 AND #4	**642**

**Embase**	**2022.03.27**	#1 'dementia'/exp OR 'Alzheimer disease'/exp OR 'mild cognitive impairment'/exp OR 'cognitive defect'/exp<br>#2 dementia OR Alzheimer OR amnestic OR amnesia OR 'mild cognitive impairment' OR ‘cognitive dysfunction':ti,ab<br>#3 'photobiomodulation'/exp OR 'phototherapy'/exp OR 'near infrared'/exp OR 'light emitting diode'/exp<br>#4 Photobiomodulation OR phototherapy OR photoneuromodulation OR photo-neuro-modulation OR 'light therapy' OR 'near infrared' OR 'near-infrared' OR laser OR 'light emitting diode':ti,ab<br>#5 'randomized controlled trial'/exp OR 'controlled study'/exp<br>#6 randomised OR randomized OR randomly OR placebo OR 'double-blind' OR trial OR groups OR 'controlled study' OR RCT OR 'single-blind*':ti,ab<br>#7 #1 OR #2<br>#8 #3 OR #4<br>#9 #5 OR #6<br>#10 #7 AND #8 AND #9<br>#11 #10 NOT ('conference abstract'/it OR 'conference paper'/it OR 'conference review'/it OR 'editorial'/it OR 'review'/it)<br>#12 #11 NOT ('animal cell'/de OR 'animal experiment'/de OR 'animal model'/de OR 'animal tissue'/de OR 'case control study'/de OR 'case report'/de OR 'ex vivo study'/de OR 'hippocampal neuronal culture'/de OR 'human cell'/de OR 'human tissue'/de OR 'in vitro study'/de OR 'meta-analysis'/de OR 'mouse model'/de OR 'nerve cell culture'/de OR 'nonhuman'/de OR 'systematic review'/de)	**637**

**CENTRAL (Cochrane Library)**	**2022.03.27**	#1 MeSH descriptor: [Dementia] explode all trees<br>#2 MeSH descriptor: [Alzheimer Disease] explode all trees<br>#3 MeSH descriptor: [Cognitive Dysfunction] explode all trees<br>#4 (dementia OR Alzheimer OR amnestic OR amnesia OR "mild cognitive impairment" OR "cognitive dysfunction"):ti,ab,kw in Trials (Word variations have been searched)<br>#5 MeSH descriptor: [Phototherapy] explode all trees<br>#6 (photobiomodulation OR phototherapy OR photoneuromodulation OR photo-neuro-modulation OR "light therapy" OR "near infrared" OR "near-infrared" OR laser OR "light emitting diode"):ti,ab,kw in Trials (Word variations have been searched)<br>#7 #1 OR #2 OR #3 OR #4<br>#8 #5 OR #6<br>#9 #7 AND #8 in Trials	**252**
**CINAHL (EBSCOhost)**	**2022.03.27**	S1 (MH “Dementia”)<br>S2 (TI dementia OR Alzheimer OR amnestic OR amnesia OR "mild cognitive impairment" OR "cognitive dysfunction")<br>S3 (AB dementia OR Alzheimer OR amnestic OR amnesia OR "mild cognitive impairment" OR "cognitive dysfunction")<br>S4 (MH “Phototherapy”)<br>S5 (TI photobiomodulation OR phototherapy OR photoneuromodulation OR photo-neuro-modulation OR "light therapy" OR "near infrared" OR "near-infrared" OR laser OR "light emitting diode")<br>S6 (AB photobiomodulation OR phototherapy OR photoneuromodulation OR photo-neuro-modulation OR "light therapy" OR "near infrared" OR "near-infrared" OR laser OR "light emitting diode")<br>S7 (MH “Randomized Controlled Trials”)<br>S8 (TI randomised OR randomized OR randomly or placebo or "double-blind" or trial OR groups OR "controlled study" OR RCT OR "single-blind*”)<br>S9 (AB randomised OR randomized OR randomly or placebo or "double-blind" or trial OR groups OR "controlled study" OR RCT OR "single-blind*”)<br>S9 S1 OR S2 OR S3<br>S10 S4 OR S5 OR S6<br>S11 S7 OR S8 OR S9<br>S12 S9 AND S10 AND S11	**109**
**PsycINFO (EBSCOhost)**	**2022.03.27**	S1 (SU dementia OR Alzheimer OR amnestic OR amnesia OR "mild cognitive impairment" OR "cognitive dysfunction")<br>S2 (TI dementia OR Alzheimer OR amnestic OR amnesia OR "mild cognitive impairment" OR "cognitive dysfunction")<br>S3 (AB dementia OR Alzheimer OR amnestic OR amnesia OR "mild cognitive impairment" OR "cognitive dysfunction")<br>S4 (SU photobiomodulation OR phototherapy OR photoneuromodulation OR photo-neuro-modulation OR "light therapy" OR "near infrared" OR "near-infrared" OR laser OR "light emitting diode")<br>S5 (TI photobiomodulation OR phototherapy OR photoneuromodulation OR photo-neuro-modulation OR "light therapy" OR "near infrared" OR "near-infrared" OR laser OR "light emitting diode")<br>S6 (AB photobiomodulation OR phototherapy OR photoneuromodulation OR photo-neuro-modulation OR "light therapy" OR "near infrared" OR "near-infrared" OR laser OR "light emitting diode")<br>S7 (SU "Randomized Controlled Trials")<br>S8 (TI randomised OR randomized OR randomly or placebo or "double-blind" or trial OR groups OR "controlled study" OR RCT OR "single-blind*")	**146**

S9 (AB randomised OR randomized OR randomly or placebo or "double-blind" or trial OR groups OR "controlled study" OR RCT OR "single-blind*")
S9 S1 OR S2 OR S3
S10 S4 OR S5 OR S6
S11 S7 OR S8 OR S9
S12 S9 AND S10 AND S11

Supplementary Table 2. Demographic and social characteristics of the included studies

Study	Country	Etiology	Severity of cognitive decline	Number of participants		Mean Age (years, SD or range)		Sex (male:female)		Mean education level (years, SD)		Adverse events	Dropouts
				Exp	Ctr	Exp	Ctr	Exp	Ctr	Exp	Ctr		
Berman et al, 2017	US	AD	Not specified	8	3	81.8 (6.3)		5:6		15.8 (2.6)	15.8 (2.6)	No reports	2
Burns et al, 2009	UK	AD, VD, LBD & mixed	Not specified	22	26	84.5 (8.0)	82.5 (7.6)	6:16	10:16	No details		Ctr: recalling unpleasure memory (1)	2
Chan et al, 2021	China	Not specified	Mild	9	9	66.4 (6.9)	66.2 (9.1)	3:6	6:3	12.1 (3.5)	11.2 (5.5)	No reports	No reports
Chao et al, 2019	US	AD	Mild and moderate	4	4	80.5 (6.5)	79.0 (5.9)	1:3	2:2	18.5 (1.9)	18.0 (1.6)	No reports	No reports
Cremascoli et al, 2022	Italy	AD	Mild and moderate (MMSE:16 -24)	8	5	72 (range 69–76)	76 (range 71–80)	6:2	3:2	No details		Ctr: ocular irritation and Burning (1)	1
Graf et al, 2001	Austria	AD & VD	MMSE≤23	13	10	78.8 (8.4)	85.8 (4.5)	No details		No details		Exp: mild conjunctivit is (1)	5 in total: adverse events (1); noncompliance (4)
Guo et al, 1998	China	VD	Not specified	26	11	67.5 (8.9)	63.9 (8.2)	16:10	6:5	No details		No reports	No reports

Huang et al, 2015	China	AD	Mild and moderate	93	34	· 30min: 71.7 (4.2), · 60min: 71.6 (3.9), · 120min: 73.3 (4.2)	72.4 (4.2)	58:69		3.8 (3.6)		Ctr: irritable (2), headache (2), ocular distention (1), and eyestrain (1)	No reports
Kim et al, 2021	South Korea	AD	Mild and moderate (CDR:0.5-2.0)	14	11	77.36(5.79)	78.55(7.71)	2:12	5:6	5.1 (3.8)	5.8 (4.0)	No reports	4(Exp:3;Ctr:1)
Liu et al, 2021	Taiwan, China	Not specified	Not mentioned	17	18	Range 60-95		1:16	6:12	Range from literate to university		No reports	No reports
Nagy et al, 2021	Egypt	AD	Mild (MoCA-B: 19-25)	30	30	69.5 (range 72-68)	70.0 (range 67-72)	30:30		Elementary education		No reports	8 (Exp: 3; Ctr: 5)
Nizamutdinov et al, 2021	US	Not specified	Mild and moderate	40	20	72.4 (8.2)	77.8 (5.2)	23:17	11:09	No details		No reports	3 (Exp:2; Ctr: 1)
Riemersma et al, 2008	Netherlands	AD, VD, LBD, mixed & other	Not specified	98	45	85 (6)	85 (5)	6:92	5:40	No details		No reports	No reports

Abbreviations: AD, Alzheimer's disease; Ctr, control group; CDR, Clinical Dementia Rating Scale; Exp, experimental group; LBD, Lewy body dementia; MoCA-B, Montreal Cognitive Assessment Scale-Basic; VD, vascular dementia.

Supplementary Table 3. Intervention-related features of the included studies

Study	Characteristics of light source			Phototherapy-related features					Accompanied therapy	
	Subtype of light	Parameters of light	Stimulation position	Length of total period	Number of sessions	Frequency	Duration of each session	Other activities during sessions	Type of accompanied therapy	Duration of each session
Berman et al, 2017	NIR LED	1068-1080 nm (1072 nm); 10 Hz	Transcranial	4 weeks	28	7/week	6min	No details	No details	No details
Burns et al, 2009	Bright light	Full spectrum (10000 lux) vs. DLT (100 lux)	Sitting in front of the light box	2 weeks	14	7/week	120min	Sitting; engaged in conversation with nurse	No details	120min
Chan et al, 2021	NIR LED	810 nm; 20 mW/cm2	Transcranial (frontal)	1 day	1	No details	350s	No details	No details	350s
Chao et al, 2019	NIR LED	810 nm; 40 Hz; 100/75/25 mW/cm2 for posterior/anterior/ intranasal respectively	Transcranial (posterior/anterior) & intranasal	12 weeks	36	3/week	20min	No details	Usual care	No details
Cremascoli et al, 2022	Bright light (Blue-enriched)	Active light treatment: 10000 lux; intensity 3/3; Sham light treatment: 50 lux (close to ambient light from the same device)	Eyes (wearing Luminette light glasses)	4 weeks	28	7/week	20min	No details	No details	No details
Graf et al, 2001	Bright light	3000 lux vs. DLT (100 lux)	90 cm in front of the light source and regularly glanced into it	10 days	10	10/10day	2h (5:00 p.m. to 7:00 p.m.)	Sitting; no details about specific activities	No details	2h (5:00pm to 7:00pm)
Guo et al, 1998	Laser	632.8nm (by He-Ne); 0.5 ~ 1.5mW	Intravascular irradiation	27 days	10	10/17day	90min	No details	Traditional medicine therapy	No details

Huang et al, 2015	Bright light	Full spectrum (1000 lux)	Eyes (best to the lower part of eyes)	1 month	30	30/month	30/60/120 min (8:00 a.m. to 11:00 p.m.)	No details	No details	No details
Kim et al, 2021	Bright light (Blue-enriched)	Blue-enriched bright light: 420-508nm (48%), 512-616nm (37%); 30 lx; Blue attenuating light (10 lx)	Sitting about 60 cm way from the light source	2vweeks	14	1-times/day, 7d/week	60min	Sitting, and allowed to do other activities (reading or listening to music)	No details	No details
Liu et al, 2021	Bright light	Bright light (2500 lux) vs. general lighting (114-307 lux)	Sitting on a chair approximately 1.2 m away from the artificial ambient exposure source at eye level	8 weeks	40	5/week	60min (9:00 a.m. to 10:00 a.m.)	Sitting; daily group session	Daily group session	60min
Nagy et al, 2021	Laser	Nasal probe: infra-red (non-thermal); Wrist watch: 650 nm (by GaA/As)	Intranasal & wrist (radial/ulnar artery and middle wrist acupoints)	3 months	78	2-times/day, 3d/week	30min	No details	Moderate-intensity aerobic exercise	45–60 min
Nizamutdinov et al, 2021	NIR LED	1060-1080 nm; 15000 mW, 23.1 mW/cm2	Transcranial	8 weeks	112	2-times/day, 7/week	6min	No details	No details	No details
Riemersma et al, 2008	Bright light	1000 lux	Eye level in the direction of gaze (slightly downward/light falling on the vertical plane)	3.5 years	No details	7/week	6h (9:00 a.m. to 6:00 p.m.)	No details	Melatonin 2.5mg qn	No details

Abbreviations: DLT, dim light therapy; LED, light-emitting diode; NIR, near-infrared.

Supplementary Table 4. Study design and measurement-related characteristics

Study	Study design	Diagnostic criteria	Neuropsychological Scale	Other measurement	Time of assessment
Berman et al, 2017	Double blind RCT	NIA-AA diagnostic guidelines for AD (2012)	ADAS-Cog, and MMSE	QEEG; Infrared spectroscopy	Baseline; 1-3d post
Burns et al, 2009	RCT	WHO (1993)	CMAI, MMSE, CSDD, MOUSEPAD, and CRBRS	Actigraphy	Baseline; 1w/5w post
Chan et al, 2021	RCT	NIA-AA diagnostic guidelines for AD (2011)	Short memory questionnaire, HKLLT, ROCFT, FPT, DS, STT, and Chinese CFT	fNIRS	Baseline; Immediately post
Chao et al, 2019	RCT	No details	ADAS-cog, MMSE, and NPI	MR including ASL and rs-fMRI	Baseline; Week 6 during the therapy; Immediately post-therapy
Cremascoli et al, 2022	RCT	DSM-IV	ESS, MMSE, PSQI	DLMO	Baseline; Immediately post-therapy
Graf et al, 2001	RCT	No details	MMSE	BTR	Baseline; Immediately post; 1d post
Guo et al, 1998	Controlled trial	No details	HDS	Plasma endothelin and erythrocyte deformability	Baseline; Day 6 during the therapy; 1d post
Huang et al, 2015	RCT	DSM-IV, NINCDS/ADRDA	PQSI, ESS, NPI, MMSE, and GDS	No details	Baseline; Immediately post
Kim et al, 2021	Single-blind controlled trial	DSM-V, CDR	BDS-ADL-K, CSDD-K, DSB, DSF, GDS-K, KESS, KNPI-Qs, MMSE-KC, PSQI, TMT-A, VAS-GV, VAS-GA,	DLMO, ZBI-K	Baseline; Immediately post-therapy; 4 weeks post
Liu et al, 2021	Single-blind controlled trial	DSM-V	MMSE, NPI	MMSE, NPI	Baseline; 5w/9w post

Nagy et al, 2021	RCT	MCI: MoCA-B basic 19–25	MoCA-B	Qol-AD; BMI; WHR; Hb level	Baseline; Immediately post
Nizamutdinov et al, 2021	Double blind RCT	No details	MMSE, ADAS-cog, CDT, LMTI&II, AVLT, DSF & DSB, CFT, TMT A&B, WAIS-R, DSST, and BNT	No details	Baseline; Immediately post
Riemersma et al, 2008	Double blind RCT	DSM-IV, NINCDS, ADRDA	MMSE, CSDD, PGCARS, PGCMS, MOSES, NPI, and CMAI	NI-ADL; Sleep efficiency; Sleep onset latency; Total sleep duration	Baseline; After 6w/6m/1y/18m/2y/2.5y/3y/3.5y

Abbreviations: AD, Alzheimer's disease; ADAS-Cog, Alzheimer's Disease Assessment Scale-Cognitive; ADRDA, Alzheimer's Disease and Related Disorders Association; ASL, Arterial spin-labelled perfusion; AVLT, Auditory Verbal Learning Test; BDS-ADL-K, the Korean Version of Blessed Dementia Scale-Activity of Daily Living; BMI, body mass index; BNT, Boston Naming Test; BTR, body temperature rhythm; CDR, Clinical Dementia Rating Scale; CDT= the Clinical Dementia Rating Scale;CDT, Clock Drawing Test; CFT, Category Fluency Test; CMAI, Cohen-Mansfield Agitation Inventory; CRBRS, Crichton Royal Behavior Rating Scale; CSDD, Cornell Scale for Depression in Dementia; CSDD-K, the Korean Version of the Cornell Scale for Depression in Dementia; DLMO, Dim Light Melatonin Onset; DS, Digit Span; DSB, Digit Span Backward; DSF, Digit Span Forward; DSM-IV, Diagnostic and Statistical Manual of Mental Disorders, Fourth Edition; DSM-V= Diagnostic and Statistical Manual of Mental Disorders, Fifth Edition; DSST, Digit Symbol Substitution Test; ESS, Epworth Sleepiness Scale; fNIRS, functional near-infrared spectroscopy; FPT, Five-Point Test; GDS, Global Deterioration Scale; GDS-K, Korean Version of the Geriatric Depression Scale; Hb, hemoglobin; HDS, Hasegawa Dementia Scale; HKLLT, Hong Kong List Learning Test; KESS, the Korean Version of Epworth Sleepiness Scale; KNPI-Qd, Korean Version of the Neuropsychiatric Inventory Questionnaire (distress); KNPI-Qs, the Korean Version of the Neuropsychiatric Inventory Questionnaire (severity); LMT-I, Logical Memory Test– Immediate total story unit recall; LMT-II, Logical Memory Test– Delayed total story unit recall; MCI, mild cognitive impairment; MMSE, Mini Mental Status Examination; MMSE-KC, Mini Mental Status Examination in the Korean Version of CERAD Packet; MoCA-B, Montreal Cognitive Assessment Scale- Basic; MOSES, Multi Observational Scale for Elderly Subjects; MOUSEPAD, Manchester and Oxford Universities Scale for the Psychological Assessment of Dementia; NIA-AA, National Institution Aging-Alzheimer's Association; NI-ADL, Nurse-informant Activities of Daily Living; NINCDS, National Institute of Neurological and Communicative Disorders and Stroke; NPI, Neuropsychiatric Inventory; PGCARS, Philadelphia Geriatric Centre Affect Rating Scale; PGCMS, Philadelphia Geriatric Centre Morale Scale; PQSI, Pittsburgh sleep quality index; QEEG, Quantitative electroencephalogram; Qol-AD, Quality-of-Life in Alzheimer's Disease; RCT, randomized controlled trial; ROCFT, Rey-Osterrieth Complex Figure Test; rs-fMRI, resting-state functional MRI; SMQ, Short Memory Questionnaire; STT, Shape Trail Test; TMT A and B, trail making tests A and B; VAS-GA, Visual Analogue Scale for Global Affect; VAS-GV, Visual Analogue Scale for Global Vigor; VD, vascular dementia; WAIS-R, Wechsler Adult Intelligence Scale-Revised; WHO, World Health Organization; WHR, waist-to-hip ratio; ZBI-K, the Korean Version of Zarit Burden Interview

2 Supplementary Figures

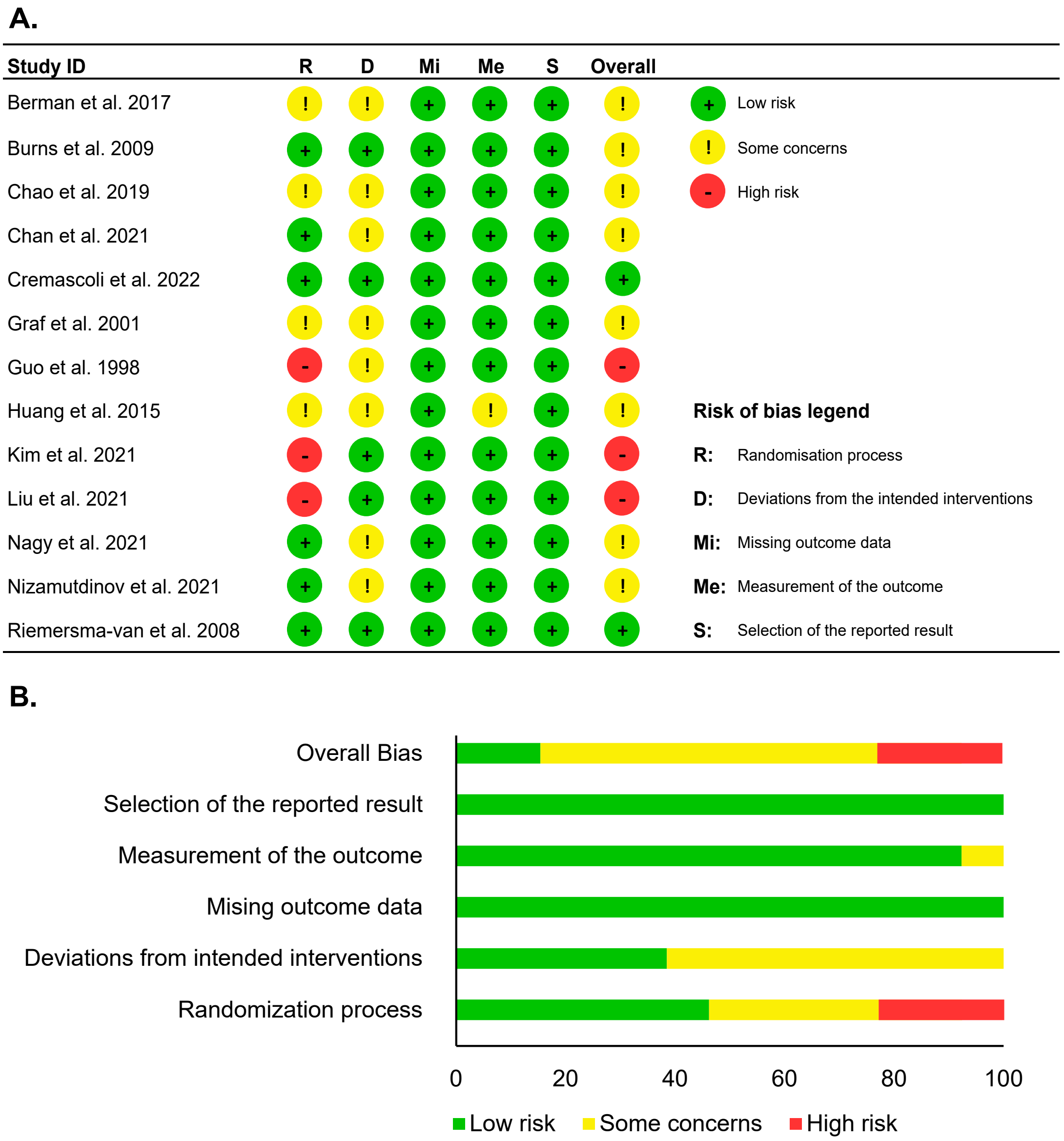

Supplementary Figure 1. Risk of bias assessment for studies using the RoB2 tool
(A) Risk of bias summary for individual studies; (B) Percentage graph of risk of bias across domains.
Abbreviation: RoB2, Risk of Bias version 2

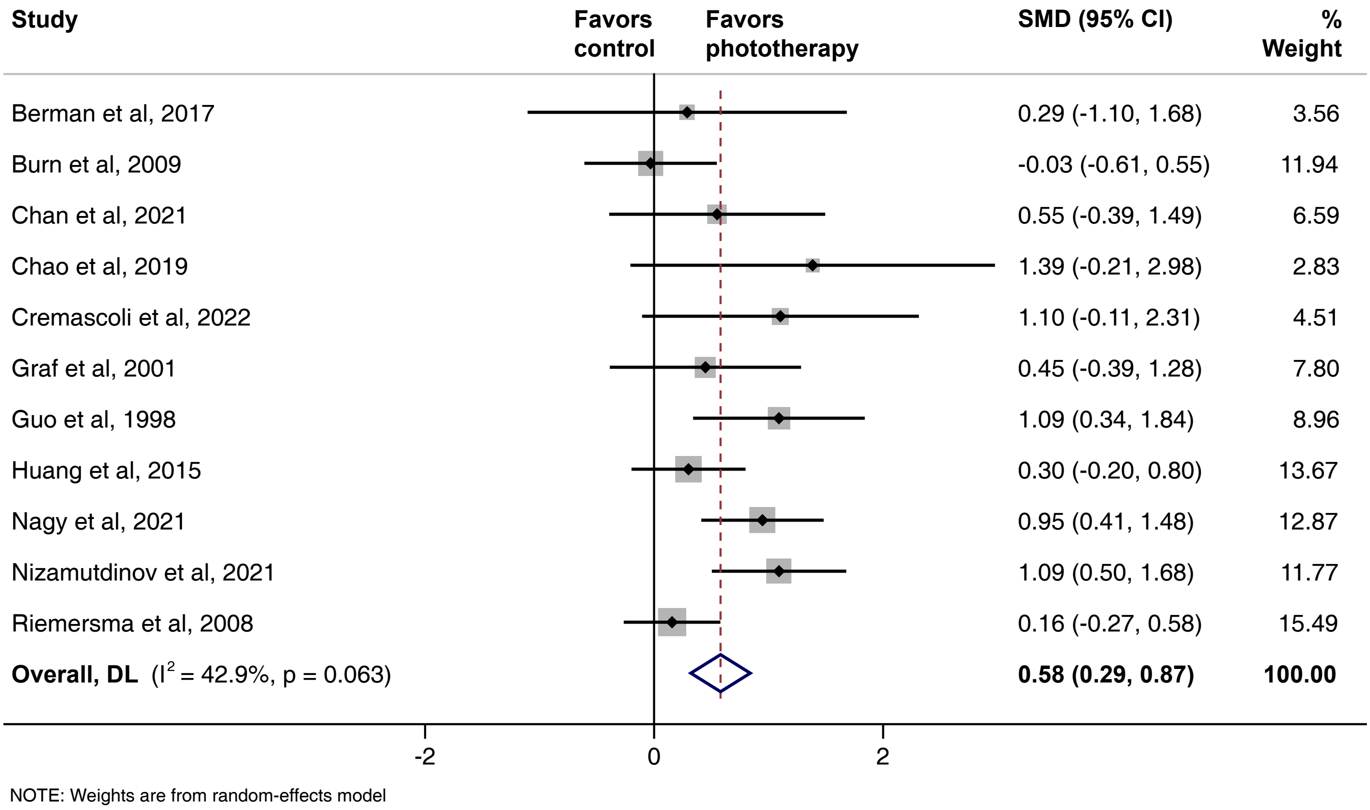

Supplementary Figure 2. Forest plot of sensitivity analysis by excluding studies with a high risk of bias. The pooled effect size measures global cognitive improvement post phototherapy compared to control.

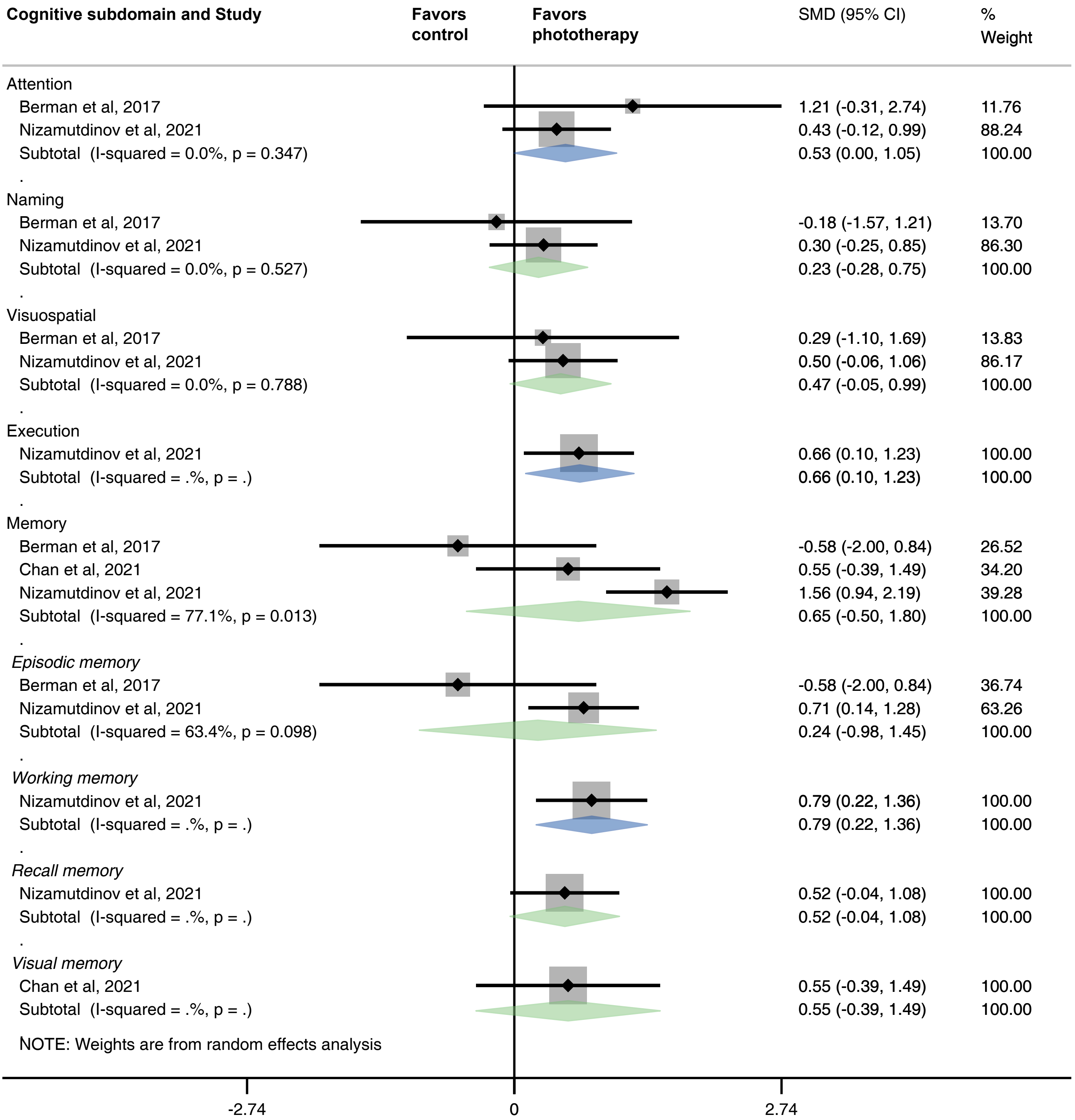

Supplementary Figure 3. Sensitivity analysis for pooled effects of different cognitive subdomains after phototherapy as compared with control interventions, by excluding studies with a high risk of bias. SMD, standardized mean difference

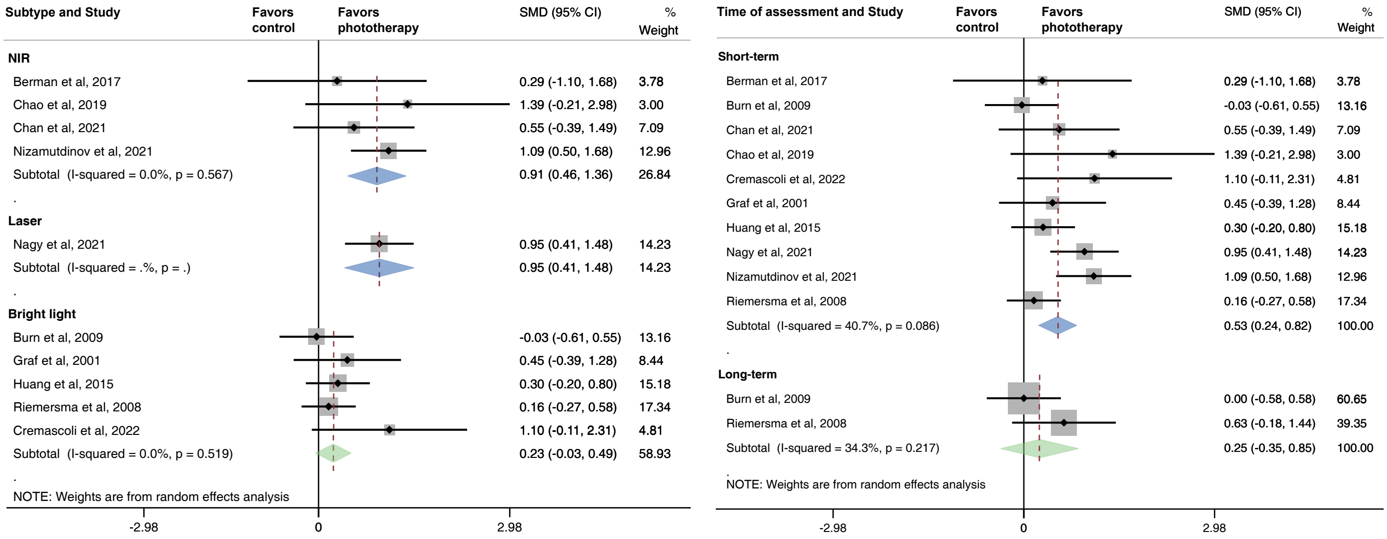

Supplementary Figure 4. Sensitivity analysis for pooled effects of subgroup analyses evaluating cognitive improvement, stratified by (A) phototherapy subtypes and (B) time of assessment.
NIR, near-infrared; SMD, standardized mean difference

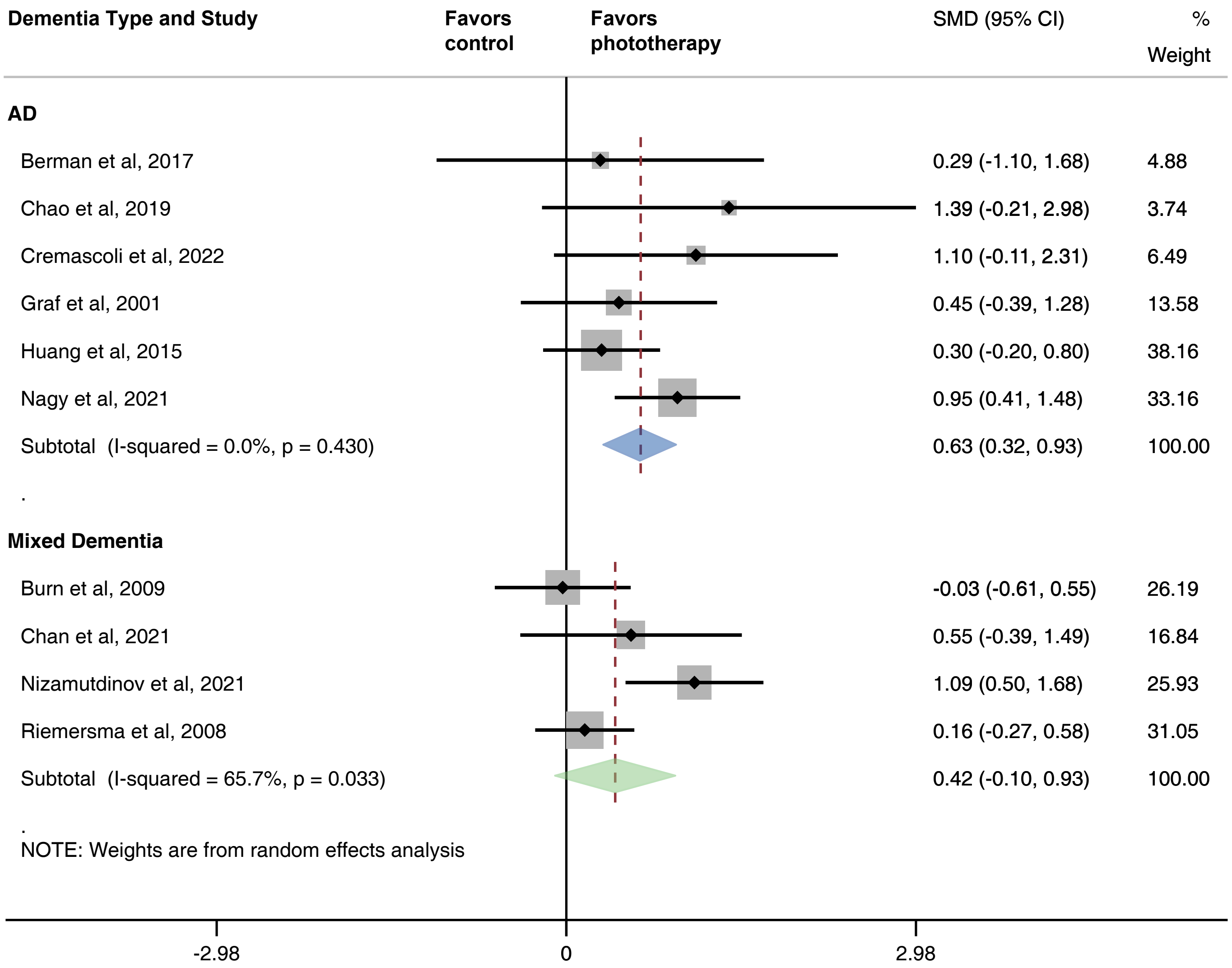

Supplementary Figure 5. Sensitivity analysis for pooled effects of subgroup analyses evaluating cognitive improvement, stratified by types of dementia.
AD, Alzheimer’s disease; SMD, standardized mean difference; VD, vascular dementia

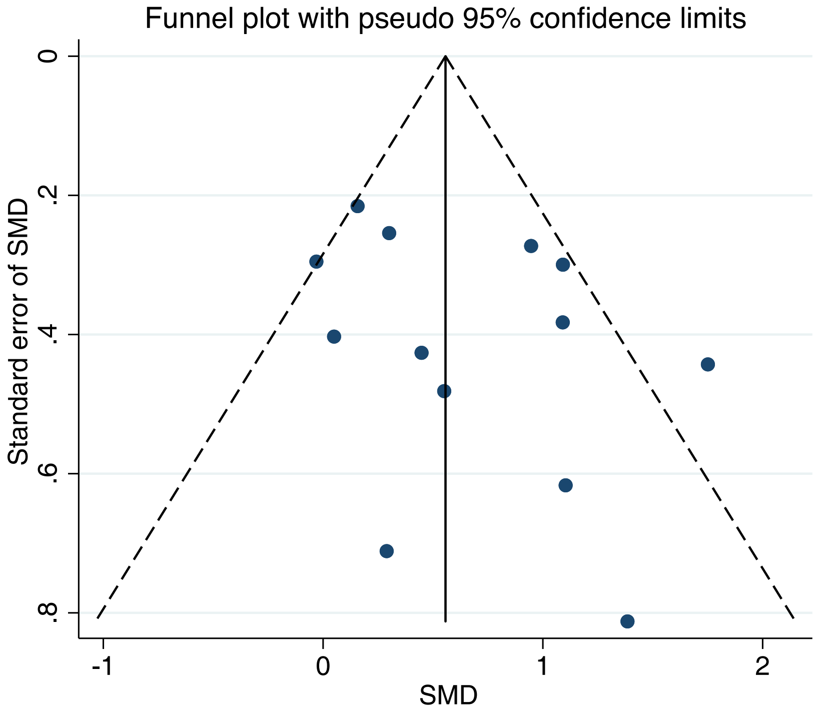

Supplementary Figure 6. Funnel plot for associations between phototherapy and global cognitive changes at the end of trials.
